# Screening of Purslane (*Portulaca oleracea* L.) Accessions for High Salt Tolerance

**DOI:** 10.1155/2014/627916

**Published:** 2014-06-09

**Authors:** Md. Amirul Alam, Abdul Shukor Juraimi, M. Y. Rafii, Azizah Abdul Hamid, Farzad Aslani

**Affiliations:** ^1^Department of Crop Science, Faculty of Agriculture, Universiti Putra Malaysia, 43400 Serdang, Selangor, Malaysia; ^2^Institute of Tropical Agriculture, Universiti Putra Malaysia, 43400 Serdang, Selangor, Malaysia; ^3^Faculty of Food Science and Technology, Universiti Putra Malaysia, 43400 Serdang, Selangor, Malaysia

## Abstract

Purslane (*Portulaca oleracea* L.) is an herbaceous leafy vegetable crop, comparatively more salt-tolerant than any other vegetables with high antioxidants, minerals, and vitamins. Salt-tolerant crop variety development is of importance due to inadequate cultivable land and escalating salinity together with population pressure. In this view a total of 25 purslane accessions were initially selected from 45 collected purslane accessions based on better growth performance and subjected to 5 different salinity levels, that is, 0.0, 10.0, 20.0, 30.0, and 40.0 dS m^−1^ NaCl. Plant height, number of leaves, number of flowers, and dry matter contents in salt treated purslane accessions were significantly reduced (*P* ≤ 0.05) and the enormity of reduction increased with increasing salinity stress. Based on dry matter yield reduction, among all 25 purslane accessions 2 accessions were graded as tolerant (Ac7 and Ac9), 6 accessions were moderately tolerant (Ac3, Ac5, Ac6, Ac10, Ac11, and Ac12), 5 accessions were moderately susceptible (Ac1, Ac2, Ac4, Ac8, and Ac13), and the remaining 12 accessions were susceptible to salinity stress and discarded from further study. The selected 13 purslane accessions could assist in the identification of superior genes for salt tolerance in purslane for improving its productivity and sustainable agricultural production.

## 1. Introduction

Salinity is regarded as a momentous situation worldwide because it has been projected that salinity will affect 30% of arable world land area within the next 25 years and about 50% of land area by the end of this century [1]. Each year, more and more land becomes nonproductive owing to salt accumulation. Salt stress can affect germination, growth, and productivity of crops as well as weeds [2]. It is now well established that salinity can affect the plant growth by altering their morphological, physiological, and biochemical as well as anatomical characteristics [3] (Tester and Davenport, 2003). Crops grown in salt affected soils may suffer from osmotic imbalance, ion toxicity, and mineral insufficiency leading to abridged growth and productivity [4, 5] and cellular dehydration is a common effect of osmotic stresses, together with water deficiency at elevated salinity levels [6, 7]. Plants grown in soils with high salt levels can also exhibit poor vegetative growth and/or symptoms with leaf sclerosis [8]. In addition, salt stress is also associated with different biotic and abiotic stresses in plants and limited their growth and development [9].

The detection of salinity-induced injuries, however, is very complex even under controlled conditions. The visual symptoms of salt stress may still be the most appropriate for mass screening. Salt injury in purslane starts with shedding of leaves and reduction of leaf area with losing of greenness and gradually stops blooming. At long time high salinity stress changing of stem color to more reddish or pinkish is also observed. The typical means of salinity tolerance is the exclusion or reduction of Na uptake and augmented assimilation of K to uphold a good Na-K equilibrium in the vegetative plant parts. Screening for salt tolerance has been undertaken worldwide using a diversity of culture techniques through plant materials ranging from germinating seeds through seedlings to mature plants in various crops by several scientists, in Jute [10], in Millet [11], in Sorghum [12, 13], in Rice [14, 15], in Pea [16], in Turfgrass [17], in Alfalfa [18], and in Wheat [2].

Cultivation of salt-tolerant species and cultivars in the salt problem soils is a substitute retrieval practice. Selection of salt-tolerant plants from salty fields or plots looks like a rational footstep for the majority plant breeders. Unluckily, the most salt-tolerant species are usually not the most productive or enviable. Due to increasing land salinization problems in the world, breeding for salinity tolerance in crops needs to pay more attention. To minimize the continuous increasing pressure of population growth on food supplies and resources, marginal land and the available water can be utilized for growing drought and saline-tolerant cultivars of different vegetables including purslane, which fills a unique and highly significant place in drought and salinity tolerance in arid zones compared to other crops [19]. The cash value of vegetable crop is always higher compared to field crops, so salt tolerance in vegetable crop is very essential [20]. Purslane is a vegetable crop species that can tolerate moderate to high salinity stress and is able to produce economic amount of dry mass even at higher salinity stress. The relative salt tolerance among different purslane cultivars has not been adequately studied yet. The proper utilization of highly salt-tolerant purslane species will give benefit to purslane growing area in Malaysia and throughout the world. So, the objectives of this study were to screen the most salt-tolerant purslane accessions prioritizing the use of this potential crop as a source of vegetable nutrients and its commercial cultivation especially for saline agriculture and sustainable development.

## 2. Materials and Methods

### 2.1. Experimental Site and Soil

The plastic pot (24 × 22 × 20 cm) experiment was conducted during April 2013 to June 2013 in the glasshouse of Field-2 at the Faculty of Agriculture, Universiti Putra Malaysia (3°00′21.34′′N, 101°42′15.06′′E, 37 m elevation). The plastic pots were filled with soil (39.51% sand, 9.03% silt, and 51.35% clay) of pH 4.8 with 2.6% organic carbon, 1.24 g cc^−1^ bulk density, and CEC of 7.07 meq+/100 g dry soil. Soil nutrient status was 0.16% total N, 5.65 ppm available P, 15.3 ppm available K, 3295 ppm Ca, and 321 ppm Mg. At field capacity, soil water retention was 31.18% (wet basis) and 45.31% (dry basis). The experimental soil belongs to the Serdang series.

### 2.2. Plant Materials and Experimental Design

Seedlings of the 12 common purslanes and cuttings of the 13 ornamental purslane accessions (as ornamental purslane does not produce seed) were selected from 45 collected purslane accessions of a previous experiment based on better vegetative growth of the plants and transplanted into the pots with prepared soils. The experiment was organized in a two-factor (purslane accessions × salinity) factorial randomized complete block design with three replications. Locations of collection and a brief phenotypic description of the 25 purslane accessions have been provided in Table 1.

### 2.3. Planting, Cultural Practices, and Treatment Application

Ten-day-old five seedlings or cuttings for each accession were transplanted in plastic pots filled with the field top soil mentioned above. The plants were allowed to recover from transplanting shock and for full establishment for 29 days. During this time, plants were irrigated with tap water as and when necessary. No fertilizer was used. Five salinity treatments (0, 10.0, 20.0, 30.0, and 40.0 Ds m^−1^) were applied in this study prepared using NaCl (Merck, Darmstadt, Germany) and distilled water. Salt treatment was initiated 30 days after transplanting (DAT) and continued till end of the study. In each pot, 200 mL of saline water was applied on alternate days according to the treatment. The control plants received 200 mL of distilled water.

### 2.4. Data Collection

#### 2.4.1. Plant Height

Sixty-day-old plant heights were measured in cm from five plants of each pot and then averaged to get the mean plant height. The mean plant height reduction due to different salinity stress was then calculated compared to untreated control plants.

#### 2.4.2. Number of Leaves

The total number of leaves of each plant was counted from each pot and averaged to calculate mean number of leaves. The mean number of shedding of leaves due to different salinity stress was then calculated compared to untreated control plants.

#### 2.4.3. Number of Flowers

Purslane blooms everyday so total numbers of flowers were counted daily and recorded. At the end before harvesting the total numbers of flowers were averaged to calculate the mean number of flowers. The mean number of flowering reduction due to different salinity stress was then calculated compared to untreated control plants.

#### 2.4.4. Total Dry Matter

For initial drying just after harvesting the fresh samples (except root) were stored in a cool dry place for 3 days, then kept in oven at 40°C temperature for 3 days (making them dry and preventing them from sudden burning injury), and then transferred to 70°C for another 72 hours to get constant weight. The mean dry weight (DW) loss due to salinity stress was then calculated from this oven-dried sample compared to untreated control plants. The percentage (%) of yield loss was measured using the following formula:

(1) Percentage(%)of  yield  loss=Control  treatment  value−Salinized  treatment  valueControl  treatment  value ×100.

Purslane accessions were classified and selected based on their total dry matter reduction due to different levels of salt impositions and were graded as tolerant (T = 0–20% reduction), moderately tolerant (MT = 21–50% reduction), moderately susceptible (MS = 51–70% reduction), and susceptible (S ≥ 70% reduction) [21].

### 2.5. Statistical Analysis

All recorded data were subjected to analysis of variance using the SAS statistical software package version 9.2 [22]. Data were submitted to analysis of variance (ANOVA) and the means were compared by Tukey's multiple range test (*P* < 0.05).

## 3. Results

**Figure 1:**
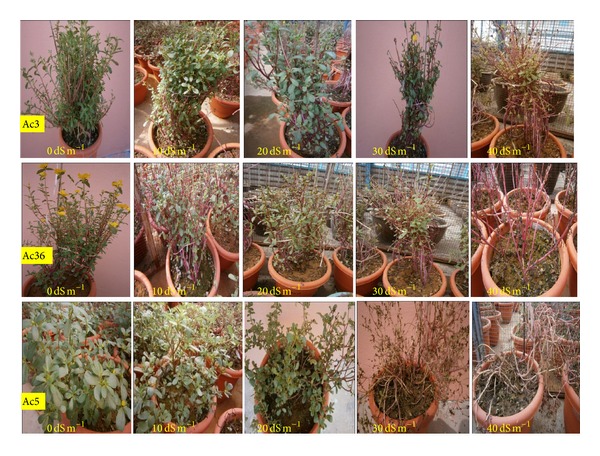
Effect of salinity on different purslane accessions.

Based on total dry matter reduction over control treatment a total of 13 purslane accessions (Ac7, Ac8, Ac36, Ac37, Ac23, Ac22, Ac24, Ac2, Ac3, Ac1, Ac4, Ac17, and Ac5) were selected from 25 accessions. Considering the above-mentioned grading classification only those accessions graded as T, MT, and MS to 30 dS m^−1^ and 40 dS m^−1^ salinity (Table 2) were selected and given chronological new accession numbers (Ac1 to Ac13) for better presentation. The other 12 accessions (graded as “S” at 30 dS m^−1^ and 40 dS m^−1^ salinity) were discarded due to very high reduction of dry matter content and their detailed analysis data have not been shown. Detailed results of selected 13 purslane accessions have been presented in Figure 1 and Tables 2–8.

### 3.1. Total Dry Matter Production

Dry matter (DM) contents in untreated control plants greatly varied (*P* < 0.001) among the 13 purslane accessions and ranged between 7.94 and 24.63 g with the highest DM content in Ac8 and the lowest in Ac5. Both of the common purslanes (Ac12 and Ac13) and 3 ornamental purslane accessions (Ac2, Ac5, and Ac10) had <15 g/pot DM contents, while the remaining eight ornamental purslane accessions produced >15 g dry matter/pot (Table 2). Salt treatment with 10 dS m^−1^ salinity significantly (*P* < 0.05) reduced DM contents in most accessions ranging between 2 and 19% with the highest dry matter loss in Ac5 (18.68%) and lowest in Ac3 (2.09%) compared to control (Table 2). However, 10–30% reductions in DM contents were recorded at 20 dS m^−1^ salinity compared to control and over 20% reductions were recorded in Ac1, Ac2, Ac5, Ac6, Ac8, and Ac12. Salt treatment with 30 dS m^−1^ salinity caused significant (*P* < 0.05) reductions (19–45%) in DM contents in all accessions compared to control. The least affected accession was Ac7 (<20% reductions). With further increase in salt concentration DM contents continued to reduce in most accessions and at 40 dS m^−1^ salinity 36–67% reductions were recorded with the lowest reduction (36.24%) in Ac10 and the highest in Ac2 (66.67%; Table 2). The overall visual effect of salinity has been shown in Figure 1.

### 3.2. Plant Height

Plant height (Ph) in untreated control plants varied very significantly (*P* < 0.001; Table 3) among the 13 purslane accessions and ranged between 30.27 cm and 66.87 cm with the highest plant height in Ac9 and the lowest in Ac13 (Table 4). On the other hand, NaCl-induced salinity also significantly (*P* < 0.001; Table 3) reduced the overall vegetative growth of purslane (Figure 1). Compared to untreated control plants, after 30 days of salt stress, plant height was highly reduced at 40 dS m^−1^ salt treatments followed by 30 dS m^−1^, 20 dS m^−1^, and 10 dS m^−1^ salt treatments, respectively (Table 4). The highest plant height reduction (>37%) was recorded at 40 dS m^−1^ salinity stress in Ac13, a common purslane, whereas the lowest plant height reduction (<4%) was observed at 10 dS m^−1^ in Ac5, an ornamental purslane (Table 4). But interestingly 2.25% increase in plant height was recorded in Ac1 at 20 dS m^−1^ salinity level compared to control. However, 8–13% reductions (*P* < 0.05) in plant height were recorded in Ac1, Ac2, Ac3, Ac8, Ac10, Ac12, and Ac13 at 10 dS m^−1^ salinity compared to control. Salt treatment with 20 dS m^−1^ salinity caused significant (*P* < 0.05) reductions (4–26%) in plant height in all accessions except Ac1 compared to control (Table 4). Rather, about 5–31% plant height reduction was noted at 30 dS m^−1^ salinity level compared to control plants. Plant height continued to reduce in most accessions with further increase in salt concentrations and 8–38% reductions were recorded at 40 dS m^−1^ salinity compared to control with the lowest reduction in Ac1 and Ac2 (<9%) and the highest in Ac13 (37.33%). On average over all accessions, 7.5, 11.5, 17.4, and 21.5% reductions in plant height were recorded, respectively, at 10, 20, 30, and 40 dS m^−1^ salinity, which were statistically significant (*P* < 0.05; Table 4).

### 3.3. Number of Leaves

Purslane is a leafy vegetable crop and it produces plentiful number of leaves. So, copping with salinity stress shedding of leaves is a major effect on purslane plants observed at different levels of NaCl-induced salinity. Untreated control plants significantly (*P* < 0.001; Table 5) varied in their mean number of leaves with the highest number in Ac13 (522.11) and the lowest in Ac12 (249.31; Table 6). Number of leaves in Ac1 and Ac8 (492.01 and 493.91) were statistically similar. Number of leaves in Ac4, Ac7, and Ac11 (465.31, 457.11, and 454.51) also were statistically similar; however, they were significantly higher (*P* < 0.05) compared to Ac3 and Ac9 (Table 6).

Salt treatment had significant (*P* < 0.001; Table 5) impact on number of leaves and responses of the 13 purslane accessions to different levels of salinity were very different from each other (Table 6). The numbers of shedding of leaves were significantly increased with the increasing of salinity stress at different levels. At 10 and 20 dS m^−1^ salinity level it was observed that shedding of leaves in Ac1, Ac2, Ac4, and Ac5 was statistically similar, whereas at 30 dS m^−1^ and 40 dS m^−1^ salinity Ac1, Ac2, and Ac4 were also statistically similar (Table 6). Furthermore, shedding of leaves was statistically similar in Ac6, Ac7, and Ac9 at 20 dS m^−1^ salinity, but at 30 dS m^−1^ and 40 dS m^−1^ the Ac10, Ac11, and Ac13 were also statistically similar (Table 6). At 10 dS m^−1^ salinity shedding of leaves ranged between 1 and 20% with the highest number of shedding in Ac1 (19.02%) and lowest in Ac11 (1.87%). And the shedding of leaves was continued to increase significantly up to 40 dS m^−1^ salinity ranging with 4–30% at 20 dS m^−1^, 7–42% at 30 dS m^−1^, and 10–47% at 40 dS m^−1^ salinity, respectively (Table 6). But in Ac5 and Ac9, all salinity levels caused significant increase in number of leaves compared to control. In Ac5 the highest increase (6.14%) of leaf numbers was observed at 10 dS m^−1^ salinity compared to control, though the percentage of increase of leaf numbers was reduced gradually to the least increase (1.66%) at 40 dS m^−1^ salinity. On the other hand in Ac9 the highest increase (9.39%) of leaf numbers was found at 20 dS m^−1^ salinity followed by 8.34% increase at 30 dS m^−1^ salinity, whereas the lowest increase (1.94%) was observed at 40 dS m^−1^ salinity followed by 2.37% at 10 dS m^−1^ salinity, respectively, compared to control by the same accession (Table 6).

### 3.4. Number of Flowers

The common purslane is very potential in blooming with only yellowish flower as well as seed production, whereas the ornamental purslane produces different colorful and attractive flowers. Salinity had a great bad impact on plants reproductive stage. Stopped blooming or shedding of flower is very common among all other morphological characteristics due to salinity stress.

Mean number of flowers in untreated control plants also significantly varied (*P* < 0.001; Table 7) between purslane accessions with the highest number in Ac12 (52.36) followed by Ac13 (46.5) and the lowest in Ac8 (4.32) followed by Ac9 and Ac11 (7.67 and 12.23). But the flower numbers in Ac6 and Ac7 were statistically similar (33.46 and 33.26; Table 8).

Salt treatments also significantly (*P* < 0.001; Table 7) impacted number of flowers in purslane plants. Treatment with 10 dS m^−1^ salinity, the Ac4 and Ac5 (Table 8), Ac10 and Ac11 (Table 8) were statistically similar (Table 8). Further at 20 dS m^−1^ salinity the Ac2 and Ac4 (Table 8) and Ac10 and Ac11 (Table 8) were also statistically similar. Statistically similar results were also observed in Ac1, Ac2, and Ac4 (Table 8), Ac8 and Ac9 (Table 8), and Ac10 and Ac11 (Table 8) at the highest 40 dS m^−1^ salinity. Flower reduction ranged between 5 and 69% at 10 dS m^−1^ with the highest number (68.18%) in Ac3 and the lowest (5.38%) in Ac7 (Table 8). At 20 dS m^−1^ salinity flower reduction observed the highest in Ac9 (83.71%) and the lowest in Ac5 (25.82%). Further, at 30 dS m^−1^ salinity flower reduction varied between 33 and 91% with the highest reduction (90.82%) in Ac1 and the lowest reduction (33.25%) observed in Ac7 (Table 8). Flower number reduction was continued significantly up to the highest level of salinity and at 40 dS m^−1^ salinity the Ac1, Ac4, Ac9, Ac10, Ac11, and Ac13 fully stopped (100%) flowering compared to control (Table 8).

## 4. Discussion

As we mentioned earlier due to high sensitivity to salinity and dry matter reduction at 30 dS m^−1^ and 40 dS m^−1^ salinity compared to untreated control (0 dS m^−1^ salinity) 13 purslane accessions (11 ornamental and 2 common purslanes) were screened out and the remaining 12 purslane accessions were discarded from detailed data presentation and discussions, that is, all thorough the paper only we have presented data of these 13 selected purslane accessions and discussed properly. However, the analysis results showed that untreated control plants greatly varied in their performance of all the recorded parameters. Salt treatment also significantly influenced all traits investigated in this study. But the responses of the 13 purslane accessions to salt treatment were very different from each other and followed a significant reduction trend from lowest to the highest salinity inductions, which indicates a vast diversity among the purslane accessions collected from different locations in Western Peninsular Malaysia.

The dry matter contents among all 13 untreated purslane accessions ranged from about 7 g to about 24 g (about 3-fold from lowest to the highest; Table 2) where Ac3, Ac8, and Ac9 were statistically similar for dry matter production (~24 g). On the other hand, Ac6 and Ac11 were also found similar (~19 g) for dry matter production but greatly varied in other parameters. Furthermore, two common purslanes (Ac12 and Ac13) were statistically different in their dry matter contents (Table 2). On the other hand, plant height ranged from about 30 cm to 67 cm (about 2-fold from lowest to highest; Table 4). The number of leaves ranged from 249 to 523 (about 2-fold from lowest to the highest, Table 6) and the number of flowers ranged from 4 to 53 (about 14-fold from lowest to the highest, Table 8).

Salt treatment also had significant impacts on plant height, number of leaves, number of flowers, and dry matter content of the 13 purslane accessions. However, responses of the individual accessions were very different from each other. One general trait was that treatment with lowest (10 dS m^−1^) to the highest (40 dS m^−1^) salinity caused significant reductions in plant height, number of leaves, number of flowers, and dry matter content.

Dry matter contents in salt treated 13 purslane accessions showed very high significant variation. From the beginning (10 dS m^−1^) to the second phase (20 dS m^−1^) increasing of salinity, the 2-fold decreasing of dry matter content was recorded in Ac1, Ac2, Ac4, Ac5, and Ac8, whereas >4-fold decrease was observed in Ac9 (Table 2). The decrease of dry matter content was less than 1-fold for other purslane accessions in that same saline condition. On the other hand, due to the increase of salinity from 20 dS m^−1^ to 30 dS m^−1^ and 30 dS m^−1^ to 40 dS m^−1^ salinity, the decreasing of dry matter content was comparatively lower than the previous state, maybe due to the increase of tolerance mechanisms among purslane accessions. Overall reductions of dry matter contents at both of this salinity phases were less than 2-fold for all the 13 purslane accessions except Ac2 and Ac7 (Table 2). The same findings of reduction of dry matter contents due to salinity stress have been described by many scientists globally in many crops. More than 70% reduction in shoot dry mass content has been reported in maize by Eker et al. [23] at 250 mM salinity. A highly significant (*P* ≤ 0.0001) decrease in both shoot and root dry matter contents was observed in sugar beet cultivars at 350 mM salinity [24]. Dry matter content of mature tomato fruits was found to be decreased with application of elevated salt treatments [25, 26]. The same results also have been reported in several crops in radish [27], in* Kyllingia peruviana* L. [28], in turfgrass species [17], in* Bruguiera gymnorrhiza* L. [29], in* Pennisetum glaucum* L. [11], and in* Brassica campestris* L. [30].

In spite of the reduction of fresh and dry matter contents many studies have also reported the positive effect of salinity stress on biomass production. Dantus et al. [31] stated the increased total biomass production in cowpea (*Vigna unguiculata* L.) seedling treated by 10 mM of sodium chloride solution. In another study, Orak and Ateş [32] and Nedjimi et al. [33] reported the increase in fresh and dry weight of shoot and root systems of common vetch (*Vicia sativa* L.) and* Atriplex halimus* L. plants treated with lower concentrations of NaCl. The increase in fresh weight may be due to the plant's ability to increase the size of its sap vacuoles, which allows for the accumulation of a lot of water, and this in turn dissolves salt ions that have amassed and leads to the subsequent augment in fresh weight [4].

Reduction in plant height is very common in many plants due to different salinity stress reported in several research articles. Though purslane is already proved as moderately tolerant to salinity, this is the first time we have observed the significant reduction in plant height at the highest salinity levels (Table 4). This result is in agreement with Yakubu et al. [34] who reported that the effect of salinity on growth of sorghum varied among the varieties. Differences in growth among* Phaseolus* species under saline condition were also reported by Bayuelo-Jiménez et al. [35]. Salinity-induced reduction in plant growth has also been reported in tomato (*Lycopersicon esculentum* Mill.) seedlings [36] and in millet seedlings [11]. The effect of salinity on growth of plants might be due to interference of nutrient absorption and physiological water stress created by high salt concentrations in the root zone [37]. It may be due to toxic effect of the NaCl used, as well as unbalanced nutrient uptake by the seedlings [38]. Decreasing trend in plant height under salinity has also been reported in many crops like in rice [39, 40], in jute [10], in* Ziziphus spina-christi* (L.) wild [41], in tomato [25], in* amaranthus* [8], in turfgrass [17], in pea [16], and in barnyardgrass (*Echinochloa crus-galli*), horse purslane (*Trianthema portulacastrum*), junglerice (*Echinochloa colona*), and rice by Chauhan et al. [42].

Purslane is a leafy vegetable crop and shedding of leaves is a major symptom due to salinity stress. It was found that the general trend of the treatment reflects a regular reduction in the number of plant leaves with the increase of salt concentration compared with the plants of the control experiment. But in Ac5 a gradual increase of number leaves was observed in all the treatment levels compared to untreated control, though the highest increase (6.14%) was observed at lowest level of salinity (10 dS m^−1^) with a consecutive decreasing of increasing rate (Table 6). However, these results of decreasing of number of leaves have been propped up by the findings of Welfare et al. [43] with their study on* Cicer arietinum* L. and López-Aguilar et al. [44] on the leaves of the tepary bean (*Phaseolus acutifolius* L.), cowpea (*Vigna unguiculata* L.), and wild bean (*Phaseolus filiformis* L). They opined that the treatment of sodium chloride reduced the number of leaves compared with control plants. Jamil et al. [45] stated that the reduction of leaf numbers on Cabbage (*Brassica oleracea* var. capitata L.) and* Brassica oleracea* var. botrytis L. is due to salinity treatments. The significant decrease in leaf number of beans (*Phaseolus vulgaris* L.) has also been reported by Gama et al. [46]. More than 50% reduction in plant height, leaf number, total leaf area, leaf chlorophyll, and dry matter content in* Ziziphus spina-christi* (L.) Willd. at 80 and 160 mM salinity has been reported by Sohail et al. [41]. The decrease of leaf numbers may be due to the accumulation of sodium chloride in the cell walls and cytoplasm of the older leaves. At the same time, their vacuole sap cannot accumulate more salt and thereby salt concentration decreases inside the cells, which ultimately leads to their quick death and cut down [4]. Uddin et al. [47] stated that the first noticed symptoms of salt overload are wilting plants and/or leaf “burn” or drying of the leaves, which are frequently originated by sodium and/or chloride toxicity. On the other hand, leaf death could be due to hastened senescence due to osmotic effect of the salt. This happens to be mostly applicable when considering the reproducibility of the responses in field condition [2].

Flowering is a life history feature resolute by plant genotype, genetic mechanism, the environment, and the interaction between them [48]. Flowering is also affected by several environmental factors such as photoperiod [49], temperature [50], herbivory [51], and water stress [52]. In our study purslane plants treated with different levels of NaCl salinity manifested significant reduction (*P* < 0.05) in flowering in both ornamental and common purslanes. The number of reduction of blooming followed the general trend of decreasing with increasing of salt concentration. Even at 30 dS m^−1^ salinity the flower reduction percentage reached 99% and the lowest was around 33% compared to untreated control. Due to further increase of salinity and at the highest level at 40 dS m^−1^ the flower reduction reached 100% for many accessions (Ac1, Ac4, Ac9, Ac10, Ac11, and Ac13); some of them (Ac2, Ac3, Ac8, and Ac12) had >90% reduction and only three purslane accessions (Ac5, Ac6, and Ac7) were able to bloom around 20–40% of flowers (Table 8). The parallel results have been reported by Zapryanova and Atanassova [53] in ornamental flowering annual species* Tagetes patula* and* Ageratum mexicanum*. On the other hand, salt-induced flowering delays have been observed in* Iris hexagona* [54], in wild mustard (*Sinapis arvensis*), an annual, nonwetland, salt sensitive species [55], and in the salt-tolerant marsh species* Cakile edentula* [56] and* Sporobolus virginicus* [57]. The inhibition of spikelet development in wheat as well as spikelet sterility in rice has also been described by Läuchli and Grattan [58].

## 5. Conclusions

Augmented tolerance to salt stress in crop plants is necessary in order to increase productivity under cropping conditions with high salinity. The present work demonstrated that under saline condition purslane accessions show substantial variation in morphological characteristics. Among the selected 13 accessions, two accessions (Ac7 and Ac9) were quite able to produce satisfactory amount of dry matter with only 0–20% reductions even at the highest 30 and 40 dS m^−1^ salinities and were graded as tolerant (T); six accessions were graded as moderately tolerant (MT; Ac3, Ac5, Ac6, Ac10, Ac11, and Ac12) with 21–50% dry matter reduction; and five accessions were moderately susceptible (MS; Ac1, Ac2, Ac4, Ac8, and Ac13) with 51–70% dry matter reduction. Considering salinity effect on plant height, Ac5 was the least affected while Ac1 showed increase in plant height at moderate salinity (20 dS m^−1^), whereas Ac5 and Ac9 were very tolerant to salinity and produced increased number of leaves at all the salinity levels compared to control. Regarding flowering Ac5 and Ac7 were able to bloom about 30% even at the highest salinity stress. So, among all 13 purslane accessions Ac5, Ac7, and Ac9 were the highest salt-tolerant accessions considering all the parameters evaluated. It was also found that the ornamental purslane showed more salt tolerance than common purslane. We hope that our findings will be very helpful for selecting purslane cultivars for commercial cultivation to fulfill the increased demands of fresh vegetables and for sustainable agriculture especially for saline areas.

## Figures and Tables

**Table 1:** Brief morphological descriptions and collection details of 25 selected purslane samples.

Accession number	Sample code	State	Locations	Latitude (°N)	Longitude (°E)	Brief morphology of the collected purslane plants
Ac1	Slg-1	Selangor	Sungai Buloh	03°19′	101°59′	Pink flower, wedge shaped margin red green leaf, red stem
Ac2	Slg-2	Selangor	Sungai Buloh	03°19′	101°59′	White-pink colored flower, wedge shaped green leaf, red stem
Ac3	Slg-3	Selangor	Sungai Buloh	03°19′	101°59′	Yellow flower, paddle shaped green leaf, red stem
Ac4	Slg-4	Selangor	AgroBio. UPM	02°98′	101°73′	Yellow flower, red margin wedge shaped green leaf, red stem
Ac5	Slg-5	Selangor	UPM	03°01′	101°71′	Wild, yellow flower, wedge shaped green leaf, green-red stem
Ac7	Slg-7	Selangor	Tanjung Karang	03°41′	101°19′	Yellow flower, paddle shaped green leaf, red stem.
Ac8	Slg-8	Selangor	Tanjung Karang	03°41′	101°19′	Pink flower, paddle shaped green leaf, red stem.
Ac9	Slg-9	Selangor	Nursery, Klang	03°02′	101°26′	Purple flower, wedge shaped red-green leaf, pink stem
Ac10	Slg-10	Selangor	Nursery, Klang	03°02′	101°26′	Pink flower, wedge shaped green leaf, green-red stem
Ac12	Mlk-1	Melaka	Kg. Pulau Gadong	02°24′	102°21′	Wild, yellow flower, wedge shaped green leaf, red-green stem
Ac17	PD-1	Negeri Sembilan	Kg. Ayer Meleleh	02°54′	101°80′	Wild, yellow flower, paddle shaped green leaf, red-green stem
Ac22	Kdh-1	Kedah	Nursery, Kedah	06°11′	100°37′	Orange-yellow flower, wedge shaped green leaf, red stem
Ac23	Kdh-2	Kedah	Nursery, Kedah	06°11′	100°37′	Pink flower, wedge shaped green leaf, red stem
Ac24	Kdh-3	Kedah	Nursery, Kedah	06°11′	100°37′	Purple flower, paddle shaped green leaf, red stem
Ac25	Kdh-4	Kedah	Kuala Kedah	06°11′	100°29′	Wild, yellow flower, green wedge shaped leaf, green stem
Ac27	Kdh-6	Kedah	Jitra-1	06°24′	100°43′	Wild, yellow flower, green wedge shaped leaf, green-red stem
Ac31	Prk-1	Perak	Kuala Kangsar	04°77′	100°94′	Wild, yellow flower, wedge shaped green-red leaf, red stem
Ac32	Prk-2	Perak	Ipoh	04°77′	100°95′	Wild, yellow flower, wedge shaped green-red leaf, red stem
Ac33	Prk-3	Perak	Perak Tengah	04°36′	100°98′	Wild, yellow flower, wedge shaped green leaf, green-red stem
Ac34	Prk-4	Perak	Bota Perak	04.34′	100°88′	Wild, yellow flower, wedge shaped green leaf, red stem
Ac35	Prk-5	Perak	Teluk Intan	04°02′	101°02′	Wild, yellow flower, wedge shaped green-red leaf, red stem
Ac36	Png-1	Penang	Seberang Perai	05°54′	100°47′	Yellow flower, paddle shaped, margin green-red leaf, red stem leaf
Ac37	Png-2	Penang	Seberang Perai	05°54′	100°47′	Pink flower, wedge shaped green red leaf, red stem
Ac38	Png-3	Penang	Seberang Perai	05°54′	100°47′	Wild, yellow flower, wedge shaped green-red leaf, red stem
Ac44	Pls-4	Penang	Kuala Perlis	06°23′	100°82′	Wild, yellow flower, wedge shaped green leaf, red stem

**Table 2:** Influence of salinity on total dry matter production of purslane plants and their classification to salinity tolerance.

Aaccession number	New acc. number	Total dry matter (g)					Classification			
		Salinity level (dS m^−1^)					Salinity level (dS m^−1^)			
		0	10	20	30	40	10	20	30	40
Ac7	Ac1	16.27^de^	14.13^de^ (13.15)	11.48^ef^ (29.44)	9.79^de^ (39.83)	5.84^fg^ (64.11)	T	MT	MT	MS
Ac8	Ac2	13.11^e–g^	11.34^ef^ (13.5)	9.67^gh^ (26.24)	8.91^d–f^ (32.04)	4.37^gh^ (66.67)	T	MT	MT	MS
Ac36	Ac3	23.55^ab^	20.38^ab^ (13.46)	18.95^b^ (19.53)	15.32^a^ (34.94)	13.32^a^ (43.44)	T	T	MT	MT
Ac37	Ac4	15.55^de^	13.97^de^ (10.16)	12.51^f^ (19.55)	9.95^d^ (36.01)	6.23^f^ (59.94)	T	T	MT	MS
Ac23	Ac5	7.94^h^	6.8^g^ (14.36)	5.71^i^ (28.08)	4.4^g^ (44.58)	3.38^h^ (57.43)	T	MT	MT	MT
Ac22	Ac6	20.67^bc^	17.77^bc^ (14.03)	15.38^cd^ (25.59)	13.02^bc^ (37.01)	9.55^bc^ (53.79)	T	MT	MT	MT
Ac24	Ac7	15.91^de^	14.17^de^ (10.93)	12.9^ef^ (18.92)	12.75^c^ (19.86)	7.98^c–e^ (49.84)	T	T	T	MT
Ac2	Ac8	24.63^a^	21.66^a^ (12.06)	17.57^bc^ (28.66)	15.16^ab^ (38.45)	8.92^cd^ (63.78)	T	MT	MT	MS
Ac3	Ac9	23.9^ab^	23.4^a^ (2.09)	21.3^a^ (10.87)	15.5^a^ (35.15)	13.48^a^ (43.59)	T	T	T	MT
Ac1	Ac10	10.1^gh^	8.6^fg^ (14.85)	8.62^h^ (14.65)	7.72^ef^ (23.56)	6.44^ef^ (36.24)	T	T	MT	MT
Ac4	Ac11	18.84^cd^	16.11^cd^ (14.49)	15.1^de^ (19.85)	13.43^a–c^ (28.72)	10.66^b^ (43.42)	T	T	MT	MT
Ac17	Ac12	14.41^ef^	13.09^de^ (9.16)	10.72^f–h^ (25.61)	9.46^de^ (34.35)	7.33^d–f^ (42.19)	T	MT	MT	MT
Ac5	Ac13	11.67^fg^	9.49^gh^ (18.68)	9.35^gh^ (19.88)	7.41^f^ (38.51)	4.04^h^ (58.17)	T	T	MT	MS

Ac9	All are discarded due to susceptible to salinity	13.44	8.32	4.11	2.01	1.8	MT	MS	S	S
Ac25		10.88	7.91	3.38	2.11	1.62	MT	MS	S	S
Ac10		16.04	10.13	4.21	2.78	2.01	MT	MS	S	S
Ac38		11.11	7.48	4.11	2.21	1.54	MT	MS	S	S
Ac32		19.23	13.09	7.21	3.33	2.07	MT	MS	S	S
Ac31		17.65	12.26	8.48	3.26	2.19	MT	MT	S	S
Ac27		21.02	14.65	8.33	2.38	1.89	MT	MS	S	S
Ac12		13.21	7.21	5.03	2.45	1.67	MT	MS	S	S
Ac33		22.04	15.28	7.11	3.21	2.06	MT	MS	S	S
Ac34		11.89	8.24	4.18	2.03	1.56	MT	MS	S	S
Ac35		8.2	4.11	2.53	1.98	1.4	MT	MS	S	S
Ac44		10.49	6.65	3.12	1.98	1.29	MT	MS	S	S

Values followed by different letters differ significantly according to Tukey's multiple range tests at *P* < 0.05. Values in the parentheses indicate percent compared to the untreated control  (0 dS m^−1^) plants.

**Table 3:** ANOVA table for plant height with salinity treatments.

Source	DF	ANOVA SS	Mean square	*F* value	Pr > *F*
Accessions	12	13730.12800	1144.17733	2087.86	<0.0001
NaCl	4	2370.54851	592.63713	1081.42	<0.0001
blk	2	5.00449	2.50224	4.57	0.0121
Accessions ∗ NaCl	48	725.39815	15.11246	27.58	<0.0001

**Table 4:** Effect of salinity on plant height of 13 salt treated purslane accessions.

Accession number	Plant height (cm)				
	Salinity level (dS m^−1^)				
	0	10	20	30	40
Ac1	39.97 ± 1.40^ab^	35.17 ± 1.60^h^ (12.01)	40.87 ± 1.60^e^ (−2.25)	33.57 ± 1.40^f^ (16.01)	36.57 ± 1.30^ef^ (8.51)
Ac2	39.67 ± 3.30^ab^	36.07 ± 0.27^h^ (9.07)	34.27 ± 0.28^g^ (13.61)	37.37 ± 0.37^e^ (5.79)	36.47 ± 0.18^ef^ (8.07)
Ac3	42.67 ± 0.55^de^	38.97 ± 0.21^g^ (8.67)	38.57 ± 0.39^f^ (9.61)	37.67 ± 0.31^e^ (11.72)	34.57 ± 0.43^f^ (18.98)
Ac4	44.97 ± 0.27^ab^	42.37 ± 0.28^f^ (5.78)	39.57 ± 0.11^ef^ (12.01)	38.27 ± 0.28^e^ (14.89)	37.37 ± 0.39 (16.9)
Ac5	39.57 ± 0.24^cd^	38.17 ± 0.21^g^ (3.54)	37.87 ± 0.07^f^ (4.29)	36.67 ± 0.39^e^ (7.33)	34.27 ± 0.19^f^ (13.39)
Ac6	56.47 ± 0.21^bc^	53.57 ± 0.23^c^ (5.14)	50.27 ± 0.18^b^ (10.98)	47.07 ± 0.31^b^ (16.65)	43.27 ± 0.21^b^ (23.38)
Ac7	53.27 ± 0.27^ab^	49.27 ± 0.25^d^ (7.51)	46.67 ± 0.31^c^ (12.38)	43.57 ± 0.35^c^ (18.21)	40.07 ± 0.16^c^ (24.78)
Ac8	49.77 ± 0.23^ab^	45.07 ± 0.39^e^ (9.44)	44.37 ± 0.27^d^ (10.84)	42.67 ± 0.31^cd^ (14.27)	40.97 ± 0.29^bc^ (17.68)
Ac9	66.87 ± 0.19^de^	64.27 ± 0.27^a^ (3.89)	60.67 ± 0.27^a^ (9.27)	54.47 ± 0.21^a^ (18.54)	50.17 ± 0.18^a^ (24.97)
Ac10	56.07 ± 0.29^d^	51.27 ± 0.24^c^ (8.56)	48.67 ± 0.33^b^ (13.19)	41.17 ± 0.17^d^ (26.57)	39.67 ± 0.33^cd^ (29.25)
Ac11	40.67 ± 0.28^ab^	38.47 ± 0.13^g^ (5.41)	35.27 ± 0.21^g^ (13.28)	33.77 ± 0.19^f^ (16.97)	30.57 ± 0.13^g^ (24.83)
Ac12	42.17 ± 0.99^e^	38.07 ± 1.09^g^ (9.72)	33.57 ± 0.84^g^ (20.39)	30.27 ± 1.19^g^ (28.22)	29.67 ± 1.13^g^ (29.64)
Ac13	30.27 ± 0.94^a^	26.47 ± 0.91^i^ (12.55)	22.57 ± 0.89^h^ (25.44)	21.10 ± 0.52^h^ (30.29)	18.97 ± 1.97^h^ (37.33)

Mean	46.34 ± 9.72^a^	42.86 ± 9.76^b^ (7.5)	41.02 ± 9.42^c^ (11.5)	38.28 ± 8.17^d^ (17.4)	36.35 ± 7.49^e^ (21.54)

Mean values with ±SE followed by different letters differ significantly according to Tukey's multiple range tests at *P* < 0.05. Values in the parentheses indicate percent compared to the untreated control (0 dS m^−1^) plants.

**Table 5:** ANOVA table for number of leaves with salinity treatments.

Source	DF	ANOVA SS	Mean square	*F* value	Pr > *F*
Accessions	12	736603.2666	61383.6055	113.57	<0.0001
NaCl	4	302386.3625	75596.5906	139.87	<0.0001
blk	2	2068.5255	1034.2627	1.91	0.1517
Accessions ∗ NaCl	48	184583.3601	3845.4867	7.12	<0.0001

**Table 6:** Effect of salinity on number of leaves of 13 salt treated purslane accessions.

Accession number	Number of leaves				
	Salinity level (dS m^−1^)				
	0	10	20	30	40
Ac1	492.01 ± 46.41^ab^	398.41 ± 32.59^bc^ (19.02)	348.11 ± 35.81^c^ (29.25)	317.41 ± 12.47^b^ (35.49)	300.32 ± 21.79^cd^ (38.96)
Ac2	467.91 ± 12.93^ab^	380.51 ± 18.55^cd^ (18.68)	329.11 ± 26.93^c^ (29.66)	318.51 ± 23.59^b^ (31.93)	300.01 ± 18.33^cd^ (35.88)
Ac3	317.51 ± 23.37^de^	280.91 ± 12.72^ed^ (11.53)	227.51 ± 24.53^d^ (28.35)	187.08 ± 72.59^c^ (41.08)	208.01 ± 27.34^ef^ (34.49)
Ac4	456.31 ± 42.78^ab^	384.21 ± 27.44^cd^ (15.81)	370.01 ± 20.33^bc^ (18.91)	316.61 ± 11.59^b^ (30.62)	288.41 ± 32.01^cd^ (36.79)
Ac5	372.51 ± 18.32^cd^	384.21 ± 15.92^cd^ (−6.14)	378.41 ± 16.48^bc^ (−3.97)	387.31 ± 12.71^ab^ (−3.14)	395.41 ± 16.58^a^ (−1.66)
Ac6	423.51 ± 14.89^bc^	394.41 ± 16.42^bc^ (6.87)	378.51 ± 15.62^bc^ (10.63)	376.41 ± 21.45^ab^ (11.12)	377.91 ± 27.49^ab^ (10.77)
Ac7	457.11 ± 24.15^ab^	410.91 ± 20.55^bc^ (10.11)	380.01 ± 22.57^bc^ (16.87)	355.41 ± 8.45^ab^ (22.25)	316.41 ± 21.42^b–d^ (30.78)
Ac8	493.91 ± 22.42^ab^	477.81 ± 28.42^a^ (3.26)	456.41 ± 23.42^a^ (7.59)	416.01 ± 15.69^a^ (15.77)	398.21 ± 16.31^a^ (19.38)
Ac9	320.31 ± 11.18^de^	327.91 ± 16.96^de^ (−2.37)	350.41 ± 13.46^c^ (−9.39)	326.51 ± 27.02^b^ (−1.94)	347.01 ± 17.85^a–c^ (−8.34)
Ac10	339.31 ± 19.18^d^	330.11 ± 10.99^de^ (2.71)	323.41 ± 13.54^c^ (4.69)	315.01 ± 18.51^b^ (7.16)	300.11 ± 23.16^cd^ (11.55)
Ac11	454.51 ± 22.43^ab^	446.01 ± 15.63^ab^ (1.87)	420.01 ± 13.33^ab^ (7.59)	356.61 ± 38.25^ab^ (21.54)	255.11 ± 20.92^de^ (43.87)
Ac12	249.31 ± 15.73^e^	240.61 ± 13.93^f^ (3.49)	222.41 ± 18.49^d^ (10.79)	168.21 ± 14.24^c^ (32.53)	163.11 ± 23.02^f^ (34.58)
Ac13	522.11 ± 17.19^a^	428.21 ± 14.97^ab^ (17.98)	385.84 ± 14.48^bc^ (26.09)	318.19 ± 18.07^b^ (39.06)	280.11 ± 25.07^cd^ (46.35)
Mean	412.79 ± 94.13^a^	376.57 ± 66.2^b^ (8.78)	352.24 ± 66.69^c^ (14.67)	319.71 ± 70.66^d^ (28.48)	301.01 ± 67.13^e^ (27.46)

Mean values with ±SE followed by different letters differ significantly according to Tukey's multiple range tests at *P* < 0.05. Values in the parentheses indicate percent compared to the untreated control (0 dS m^−1^) plants. “−” indicates % increase due to salinity stress.

**Table 7:** ANOVA table for number of flowers with salinity treatments.

Source	DF	ANOVA SS	Mean square	*F* value	Pr > *F*
Accessions	12	11181.42225	931.78519	2058.49	<0.0001
NaCl	4	14251.59393	3562.89848	7871.12	<0.0001
blk	2	2.77409	1.38704	3.06	0.0501
Accessions ∗ NaCl	48	5435.35492	113.23656	250.16	<0.0001

**Table 8:** Effect of salinity on number of flowers of 13 salt treated purslane accessions.

Accession number	Number of flowers				
	Salinity level (dS m^−1^)				
	0	10	20	30	40
Ac1	23.54 ± 0.69^g^	15.42 ± 5.01^f^ (34.49)	6.12 ± 0.80^g^ (74.01)	2.16 ± 0.34^h^ (90.82)	0^f^ (100)
Ac2	35.22 ± 0.69^c^	28.52 ± 0.40^c^ (19.02)	16.78 ± 0.42^e^ (52.36)	12.11 ± 0.35^d^ (65.62)	0.14 ± 0.19^f^ (99.6)
Ac3	22.66 ± 0.31^g^	7.21 ± 0.16^h^ (68.18)	4.28 ± 0.22^h^ (81.11)	6.77 ± 0.23^f^ (70.12)	1.27 ± 0.23^e^ (94.39)
Ac4	32.17 ± 0.17^e^	26.02 ± 0.26^d^ (19.12)	17.49 ± 0.27^e^ (45.63)	6.69 ± 0.31^e^ (79.2)	0^f^ (100)
Ac5	27.19 ± 0.21^f^	25.71 ± 0.35^d^ (5.44)	20.17 ± 0.21^c^ (25.82)	15.26 ± 0.16^b^ (43.88)	10.03 ± 0.27^a^ (63.11)
Ac6	33.46 ± 0.19^d^	28.46 ± 0.34^c^ (14.94)	23.42 ± 0.25^b^ (30.01)	14.44 ± 0.32^c^ (56.84)	6.0 ± 0.25^f^ (82.07)
Ac7	33.26 ± 0.21^d^	31.47 ± 0.15^b^ (5.38)	26.28 ± 0.17^a^ (20.98)	22.20 ± 0.16^a^ (33.25)	8.30 ± 0.26^b^ (75.05)
Ac8	4.32 ± 0.22^k^	2.82 ± 0.20^j^ (34.72)	1.16 ± 0.22^i^ (73.15)	0.77 ± 0.21^i^ (82.18)	0.12 ± 0.12^c^ (97.22)
Ac9	7.67 ± 0.36^j^	4.01 ± 0.20^i^ (47.72)	1.28 ± 0.12^i^ (83.31)	0.08 ± 0.13^i^ (98.86)	0^f^ (100)
Ac10	14.42 ± 0.14^h^	11.02 ± 0.21^g^ (23.58)	6.70 ± 0.28^g^ (53.54)	2.13 ± 0.16^h^ (85.23)	0^f^ (100)
Ac11	12.23 ± 0.23^i^	10.47 ± 0.29^g^ (14.39)	6.58 ± 0.21^g^ (46.19)	0.17 ± 0.15^i^ (98.61)	0^f^ (100)
Ac12	52.36 ± 0.28^a^	22.42 ± 0.32^e^ (57.18)	18.57 ± 0.44^d^ (64.53)	8.31 ± 0.31^e^ (84.13)	2.36 ± 0.22^d^ (95.49)
Ac13	46.50 ± 0.22^b^	34.21 ± 0.19^a^ (26.43)	14.50 ± 0.22^f^ (68.82)	5.12 ± 0.41^g^ (99.98)	0^f^ (100)

Mean	26.54 ± 14.41^a^	19.06 ± 10.98^b^ (28.19)	12.56 ± 8.58^c^ (52.66)	7.40 ± 6.86^d^ (72.11)	2.17 ± 3.55^e^ (91.82)

Mean values with ±SE followed by different letters differ significantly according to Tukey's multiple range tests at *P* < 0.05. Values in the parentheses indicate percent compared to the untreated control (0 dS m^−1^) plants.
